# Medical Sketches Taken in the Royal Navy

**Published:** 1812-03

**Authors:** James Robinson Scott

**Affiliations:** SURGEON, R.N.


*For the Medical and Physical Journal.*

### MEDICAL SKETCHES *taken in the* ROYAL NAVY, *by* JAMES ROBINSON SCOTT, SURGEON, R.N.

(Continued from p. 94.)

### *Depletion by the Lancet.*

IN the beginning of the present year (1811), being on board H. M. hospital-ship Triton, lying in the Tagus, I was particularly struck with a case of extensive visceral inflammation, which had a fatal termination, and in which depletion had been much neglected, before the patient was brought to this vessel for relief: so much so, that the subsequent remedies, of which venesection was one, were of no use. He was brought there for the cure of thoracic inflammation, mation*, and died in exquisite agony a few days after. What the circumstances were, attendant upon this case, I could not exactly learn; a faint outline of it only, I believe, having been transmitted with him. This case made a deep impression on my mind; and that depletion had been neglected was remarked by the medical attendants, both before death and at the dissection, where I assisted, and observed on opening the thorax an uncommonly large quantity of blood extravasated in both cavities. The lungs in both sides were loaded with blood, and had suffered inflammation throughout their substance. The blood found in the cavities was of a very florid color. In the abdominal cavity the liver exhibited marks of inflammation on its surfaces; the spleen was enlarged very much, and weighed a pound and a half; it resembled a large purple inorganised mass, having suffered a complete dissolution of its vascular structure: it was not schirrous. The ilium was inflamed in several places. The patient had laboured under intermittent before he had been attacked by his other complaints: hence probably the cause of the distended spleen. His sufferings where confined almost entirely to his breast when alive. I am induced to think that the intestinal inflammation was either subsequent to, or synchronous with, the pulmonary one. The patient was opened a few hours after his death. I found on cutting into the heart, that its fibres were remarkably relaxed, and pale. Some surgeons of talent and eminence in the fleet saw this dissection, and expressed their conviction that the lancet had not been promptly used. This is one out of the many cases which I could adduce of similar procrastination.

How frequently may bleeding be used in intermittents with advantage? not directly to remove the complaint, but to exonerate the system from the load of circulating fluid, determined by each successive fit to the different viscera, more or less, according to their state of predisposition. While the system is supported by moderately nutritious diet, bleeding, at the commencement of the hot stage, when such great reaction is present, will obviate, by saving the

* I apply the term thoracic inflammation to inflammation which attacks any part of the lungs or their appendages, within the thorax, including, of course, the pleuræ and mediastina. I think the distinctions of definition, such as pleuritis, pneumonia, &c. useless, as they are so often combined, so easily confounded, and as the same treatment holds good in all. I always mean, when I use the word depletion in this essay, depletion by blood-letting. I think the term direct depletion would be very proper to characterise evacuation by the lancet.

viscera, viscera, in some measure, the danger of subsequent alarming acute or incurable chronic affections.

While I am on the subject of bleeding in thoracic inflammation, I shall make some remarks upon a mode of practice sanctioned in this disease by some, and opposed by others. I mean the exhibition of opium and calomel. I do not presume to set up my opinion on this subject, so as to offend any individual. It has been long used by some of the most respectable practitioners.

I only mean to speak of its effects as far as they have fallen under my own observation on board ship: I shall allude to my own case, and the effects of its treatment; and give my reasons for protesting against the opinion "that this mode of practice may supersede, in many cases, the use of direct depletion." This opinion, if acted upon, may be the means, I am convinced, of destroying numbers, or of shortening their lives, and making them miserable for the remainder of their days.

Let us first take a view of the *modus operandi* of that powerful stimulant, opium; inquire into its actual effects in thoracic inflammation, with or without calomel; and consider how far it is, in this instance, to be trusted. Opium first acts on the nerves of the stomach, with which it comes in contact; by them its effects are conveyed to the organ of volition, increasing its action, and thus also the action of the vascular system; the pulse becomes more quick and full, and the surface increases in its degree of temperature. Mental action is also increased.

From increased action of the brain arises diminution of power; hence succeed torpor of the sensorium; deranged state of the stomach; furred tongue; in fact, such symptoms as are produced by inebriety. This is commonly the regular succession of effects that follow the exhibition of opium, even in large doses. Stimulation precedes torpor, but in some constitutions there is certainly less than in others; and then a much smaller dose is requisite to produce the state of torpor. These effects have uniformly been produced on myself by opium, in whatever dose I have taken it; when obliged to use it at any time to allay pain or irritation, even to the extent of sixty drops of the tincture, though I might not have taken any for three months before, and am not in the habit of using it.

Where a viscus is inflamed; where a cessation of excessive vascular distention is wished for; when pain, and irritation referable to nerves, do not form the most prominent symptoms; then I should distrust the efficacy of opium.

Diseases differ from each other so much, according to their

 situation, situation, that, although many of them are classed together to assist the memory of the student, and simplify the science, yet this classification ought not to bias us in our particular treatment of those diseases. Opium may be exceedingly safe and efficacious in acute rheumatism (and, that it is, has been abundantly testified), yet it may also be neither very safe nor very efficacious in inflammation of the lungs or stomach.

Dr. Hamilton used venesection with his opium. Let us combine opium with calomel, tartar emetic, and camphor:— Tart. emetic is a diaphoretic, camphor allays excessive action, and calomel may be useful in two ways,—by determining to the salivary glands; by increasing other secretions and excretions, and thus lessening the determination to the lungs; otherwise it is an improper stimulus to an inflamed viscus. An experienced and intelligent surgeon, of eighteen years' practice in the navy, with whom I conversed relative to this practice, tells me that he has used Dr. Hamilton's plan with advantage; but he bled very copiously at the same time. I have seen opium, when used *with* V. S. do harm. No practitioner is safe in trusting either to it or calomel, or both conjoined, in pulmonary inflammation, *without* the use of the lancet.

Its advocates say, that opium in thoracic inflammation is not so useful by itself as when conjoined with other medicines. The effects of these separately are known: conjoined with depletion by the lancet, any one of them is better, as an auxiliary, than opium. Opium is useful in rheumatism and dysentery *by itself*. But, if it be injurious in thoracic inflammation by itself, its junction with calomel cannot make it less so; unless by chemical combination it forms a new body, which is not the case. The mixture is mechanical, and the two medicines will exert separate influences on the system: thus opium will render the bowels torpid, prevent the calomel from passing away by stool, facilitate its absorption, and perhaps produce an effect on the lungs, by the nerves, so as to make them less liable to be irritated by the stimulus of calomel passing through the circulating medium.

From my own feelings while labouring under those affections, I can decidedly speak against the use of opium. I suffered much from the exhibition of this medicine. I have given my reasons for rejecting opium and calomel, either separately or conjoined. In short, calomel is an additional cause of irritation; opium increases the fullness and quickness of the pulse, causing vascular distention in the lungs; and and ought scarcely to be used even with venesection: but, shall we attempt to substitute it for this evacuation?

It is just and fair to examine into the manner in which substances act on the human system. From theory, or from practice, I can gather nothing to favor the opinion that opium may, in any instance, supersede the use of the lancet. Where I used this medicine myself, and saw it used by others, it was always when conjoined with venesection, except in my own case.

Happy would it be for suffering humanity if a specific could be found for these formidable affections; but I fear that those who dream of having discovered it in calomel and opium, to the exclusion of blood-letting, will find themselves grievously mistaken: and, if the assertion made by some with such confidence, relative to the irretrievable mischief done by copious depletion in such complaints, be true, sad were the lot of those attacked with them, and dreadful their alternative.

We may conclude, upon the whole, that it can be no very arduous task for any practitioner, by looking at his patient, interrogating him, weighing in his mind every peculiarity of constitution, and circumstance of situation, and attending to his age, to form a pretty correct decision, relative to his treatment of any case of acute visceral inflammation.

Let us proportion our evacuations to the real strength of our patient; observing that we are not imposed on by the appearance of debility, depending solely upon the disease requiring evacuation. We ought to calculate on the probability of the disease, if V. S. be neglected, producing more injury than the degree of debility, whether temporary or permanent, likely to be caused by bleeding our patient largely.

In March, 1810, while in a small gun-brig, during which time we lay in the river at Oporto, I was seized with a sudden and severe attack of pulmonary inflammation. On the first night of my illness, my pulse rose to 140, and I experienced constant danger of suffocation during the night. No vessel being then on the station near us, and as I was not immediately aware that an English physician resided in Oporto, I delayed inquiring about medical assistance during the first 24 hours, and I had an insurmountable reluctance to calling in any Portuguese practitioner, even to perform venesection. I was not so well aware of my danger as subsequent circumstances proved I ought to have been. I had had a catarrhal affection during part of the winter, and was at this time considerably emaciated. I had been exposed to wet and cold for some months, and was then lying in a very small wet cabin, in a vessel which had never been dry for months; and at a season when, in those parts, the weather is cloudy, and the rain for many weeks almost incessant. Considering these circumstances, I concluded, though erroneously, that direct depletion might too much debilitate me. Having recourse to the tincture of digitalis for the first time in my life, I took of this medicine 20 drops, conjoined with a few of laudanum, every three hours; and I took in this way upwards of 200 drops before I found my pulse lowered to 95. Perspiration coming on shortly after, I discontinued the digitalis, purged, and used large doses of pulv. ipecac. comp. My pulse continuing to rise in the evening to 120, and the symptoms of oppression at the præcordia being but little abated, I was apprehensive of the great injury which I might sustain, should I longer delay medical advice. I called in Dr. ——, resident at Oporto, some days after I had been attacked by the complaint. On my earnestly requesting him to take some blood from me, he to my surprise refused; though I dwelt much on the state of my pulse, and the uneasiness I felt in my breast. He spoke of the danger of my falling into typhus, if bled, owing to the state of the climate at that time; of my not being at all plethoric, &c. talked much of spasm, and advised the continuance of the digitalis and pulv. ipecac. comp. also calomel as a purge: he likewise talked of bile on the stomach, &c. On my persisting in being bled, he said that he would withdraw his attendance. I gave up the point, as there was no other gentleman of the profession there that I knew of, and as I then began to expectorate largely, and consequently supposed that the violence of the disease was thus about to abate.

I expectorated for three weeks so largely, that, with it, and perspirations at night, I was reduced to an alarming state of weakness. About the 1st of April I could walk about a little; but for two months afterwards, the slightest exertion would raise my pulse to 110 or 120. The inflammatory symptoms in the thorax abated in four days, but the severe ones which followed lasted some months; and, indeed for two months after the immediate danger was over, I had every apparent symptom of incipient phthisis. I have never enjoyed the same share of health since, experiencing that disagreeable train of symptoms allied to adhesions, dyspnœa, &c. Dr. ——, during my illness, paid me the most polite attention.

I look upon this case as one of spontaneous termination: the sudden appearance of copious expectoration, perspi-

 ration, ration, &c. lead me to think that the medicines which I took were of little use, except perhaps the digitalis, which I have always considered a very valuable medicine, though its worth has been often enthusiastically overrated. The pain I suffered in my breast was not severe. I could never refer it to any particular part: it seemed interwoven with the universal sense of oppression and restlessness, and a sensation of a load in the lungs; and their feeling as if impervious to the air. These feelings were different from those generally present in the spasmodic disease asthma. I suffered most from want of rest, to procure which I took laudanum, often indeed, but never with advantage; it always kept me from sleep and rest, increased the quickness of the pulse, and caused me to feel redoubled uneasiness in my breast every day which succeeded the use of it.

